# Fabrication of 9.6 V High-performance Asymmetric Supercapacitors Stack Based on Nickel Hexacyanoferrate-derived Ni(OH)_2_ Nanosheets and Bio-derived Activated Carbon

**DOI:** 10.1038/s41598-018-37566-8

**Published:** 2019-01-31

**Authors:** Subramani Kaipannan, Sathish Marappan

**Affiliations:** 1 Functional Materials Division, CSIR-Central Electrochemical Research Institute, Karaikudi, 630 003 Tamil Nadu India; 2 Academy of Scientific and Innovative Research (AcSIR), CSIR-Central Electrochemical Research Institute, Karaikudi, 630 003 Tamil Nadu India

## Abstract

Hydrated Ni(OH)_2_ and activated carbon based electrodes are widely used in electrochemical applications. Here we report the fabrication of symmetric supercapacitors using Ni(OH)_2_ nanosheets and activated carbon as positive and negative electrodes in aqueous electrolyte, respectively. The asymmetric supercapacitors stack connected in series exhibited a stable device voltage of 9.6 V and delivered a stored high energy and power of 30 mWh and 1632 mW, respectively. The fabricated device shows an excellent electrochemical stability and high retention of 81% initial capacitance after 100,000 charge-discharges cycling at high charging current of 500 mA. The positive electrode material Ni(OH)_2_ nanosheets was prepared through chemical decomposition of nickel hexacyanoferrate complex. The XRD pattern revealed the high crystalline nature of Ni(OH)_2_ with an average crystallite size of ~10 nm. The nitrogen adsorption-desorption isotherms of Ni(OH)_2_ nanosheets indicate the formation of mesoporous Ni(OH)_2_ nanosheets. The chemical synthesis of Ni(OH)_2_ results the formation of hierarchical nanosheets that are randomly oriented which was confirmed by FE-SEM and HR-TEM analysis. The negative electrode, activated porous carbon (OPAA-700) was obtained from orange peel waste. The electrochemical properties of Ni(OH)_2_ nanosheets and OPAA-700 were studied and exhibit a high specific capacity of 1126 C/g and high specific capacitance of 311 F/g at current density of 2 A/g, respectively. Ni(OH)_2_ nanosheets delivered a good rate performance and remarkable capacitance retention of 96% at high current density of 32 A/g.

## Introduction

One of the greatest hurdles which researchers face today indubitably is the development of highly efficient energy storage device with high power and energy density^1^. Such devices which are being utilized in diverse fields should possess both high energy density and fast charging/discharging ability^2–5^. Conventional capacitors are capable for fast static electric storage but exhibit relatively low energy density. While the batteries suffer from slow electrode reactions to realize relatively high energy densities^3,6^. To fill the gap between the capacitors and batteries, an electrochemical energy storage device known as electrochemical capacitors or supercapacitors have been developed. It acts as a bridge to balance energy density and power density by combining fast surface-tended electrochemical reactions and static electric storage has been developed recently by researchers^7–10^. The large specific capacitance achieved by these supercapacitors can be attributed to two different charge storage mechanisms occurring at their electrode/electrolyte interface^11,12^. The electrical double-layer capacitance (EDLC), which stores charge electrostatically or non-faradaic and it doesn’t involve transfer of charge between electrolyte and electrode. EDLC typically involves using high surface area carbon based materials like activated carbon, carbon nanotube and graphene as electrode materials^13,14^. Since EDLC does not involve charge transfer at electrode/electrolyte interface, the chance of chemical or composition changes associated with the non-faradaic process is none^15,16^. For this reason, charge storage in EDLC is highly reversible, which makes them worthy candidate for achieving very high cycling stabilities and they generally operate with stable performance characteristics for a great many charge-discharge cycles^17–19^. The other mechanism is based on the charge-transfer Faradaic reaction which stores energy by redox reactions of metal oxides/hydroxide or electrically conducting polymers which are used as electrode materials to deliver high energy density^20,21^. Depending on the type of electrode materials used in the supercapacitor, these two mechanisms may occur simultaneously or independently^22^.

Generally, metal oxides exhibit higher specific capacitances and electrochemical stability compared to most of the conductive polymers; hence they can be used as ideal electrode materials for fabricating high-energy supercapacitors^23–25^. Among metal oxide materials, RuO_2_ has been extensively explored due to its extraordinarily high specific capacitance, good conductivity and three distinct oxidation states accessible within 1.2 V^26,27^. However, because of its high cost and toxicity, RuO_2_ as an electrode material for supercapacitors in a commercial level is being lot more cogitated. Hence, transition metal (such as Fe, Co, Cu, Ni and Mn) oxides/hydroxides are under extensive investigation as alternate electrode materials for supercapacitors^15,28,29^. Among these metal oxides/hydroxide, Ni(OH)_2_ is a potential candidate due to its elemental abundance (second most abundant element in the earth’s core), non-toxicity, easy preparation in various shapes at the nanoscale with excellent electrochemical performance like high theoretical specific capacity of 1041 C/g^25^. Recently, many attempts have been made to explore Ni(OH)_2_ based electrode materials for supercapacitors applications with different surface morphologies like plates^30^, tubes^31^, flowers^32^, flakes^33^, paraotwayite^34^, hollow spheres^35^, and so on. Among the different surface morphologies, complex hierarchical structure exhibits excellent performance for supercapacitors because of their peculiar structures and utilizing both Faradic and non-Faradaic process.

To date, different methods are used to synthesize hierarchical structure Ni(OH)_2_ using hydrothermal, solvothermal and other routes^36^. All these method involves either temperature, pressure and time dependent growth of Ni(OH)_2_ which affects the overall electrochemical properties of electrode materials and struggle to scale up for large scale production and fabrication of supercapacitors. Few attempts have been made to fabricate supercapacitor stack with the series or parallel connections and bipolar stacking by connecting two or more supercapacitors to reach a maximum cell voltage and deliverable energy^37–40^. Peng *et al*., showed two laser-induced graphene supercapacitors connected in series and parallel connections with two times of higher cell voltage and longer discharge time, respectively^41^. In stacked supercapacitor assembly, when the cells are connected in series the voltage of the stack increase with decreases in the net capacitance. While, the cells are connected in parallel, the stack net capacitance will increase. Thus, combination of series and parallel stacking of supercapacitors is necessary to obtain desired working voltage with optimum stored energy. The El-Kady *et al*. proposed direct laser reduction of graphite oxide films based supercapacitor with the two cells in serial connection to get the output voltage to 2 V. Then, two cells with 2 V are connected in parallel connection to double capacitance^42^. Most recently, Wang *et al*., reported on fabrication of in-plane and multi-layer 3D micro-supercapacitors derived from a laser carbonization of polyimide sheets^43^. In this work, it was clearly justified that the combination of series and parallel (two in series and four in parallel) connection can effectively increase the overall cell voltage and energy density. In our earlier attempt, four all-solid-state symmetric supercapacitors (1 V) were connected in series that attained a cell voltage of 4 V with a high energy and power density of 22.4 Wh/kg and 2037 W/kg, respectively^13^. Similarly, all-solid-state asymmetric supercapacitor (CoS and activated carbon) was reported where the cell voltage of asymmetric supercapacitor was increased to 3.6 V by connecting two cells in series^44^. Even though, there are several papers have been reported with high specific capacitance, high energy and power density for the fabrication of supercapacitor, the actual deliverable energy and power is less due to low active mass loading. The actual stored energy (E_st_) and deliverable power (P_st_) decides the applications of fabricated supercapacitors. For high energy storage applications like electric and hybrid vehicles, the expected energy storage needs in the range of mWh to Wh. Thus, demonstration of mWh supercapacitor assembly by combination of parallel and series connection with high cell voltage with high energy storage is highly warranted.

Here, we report the synthesize of Ni(OH)_2_ nanosheets *via* a scalable and convenient route with hierarchical structure using two step chemical process. The synthesis involves the formation of NiHCF nanosphere and followed by chemical decomposition of NiHCF to Ni(OH)_2_ nanosheets. This promising simple approach for synthesize of Ni(OH)_2_ nanosheets is scalable for large scale production. The obtained mesoporous Ni(OH)_2_ nanosheets exhibit a high specific capacity of 1126 C/g at current density of 2 A/g with good rate performance of 46% (512 C/g at current density of 32 A/g). Even at 10000 cycles with high current density of 32 A/g, Ni(OH)_2_ nanosheets exhibit remarkable capacitance retention (96%). Even though electrode materials deliver extraordinary electrochemical properties, the lower working voltage (0.45 V *vs*. Hg/HgO) limits their energy density for further fabrication of supercapacitor devices. Therefore, Ni(OH)_2_ nanosheets based positive was combined with carbon based negative electrodes and a asymmetric cell was fabricated with a cell voltage in the range of 1.4 to 1.8 V. In our present report, we fabricated asymmetric supercapacitor with orange peel derived activated nanoporous carbon and NiHCF derived Ni(OH)_2_ nanosheets were used as negative electrodes and positive electrodes, respectively. The fabricated asymmetric supercapacitor in aqueous electrolyte exhibits a stable cell voltage of 1.6 V. To increase the device voltage, six cells were connected in series delivered an E_st_ and P_st_ of 30 mWh and 50 mW at 10 mA charging current, respectively. The charging current increased to 500 mA, the fabricated asymmetric supercapacitors delivers an E_st_ and P_st_ of 2.5 mWh and 1632 mW, respectively. Similarly, cycling at high charging current of 500 mA shows an 81% of initial capacitance retained even after 100,000 charge-discharge cycles which shows an excellent electrochemical stability of fabricated asymmetric supercapacitors for viable applications.

## Results and Discussion

**Figure 1:**
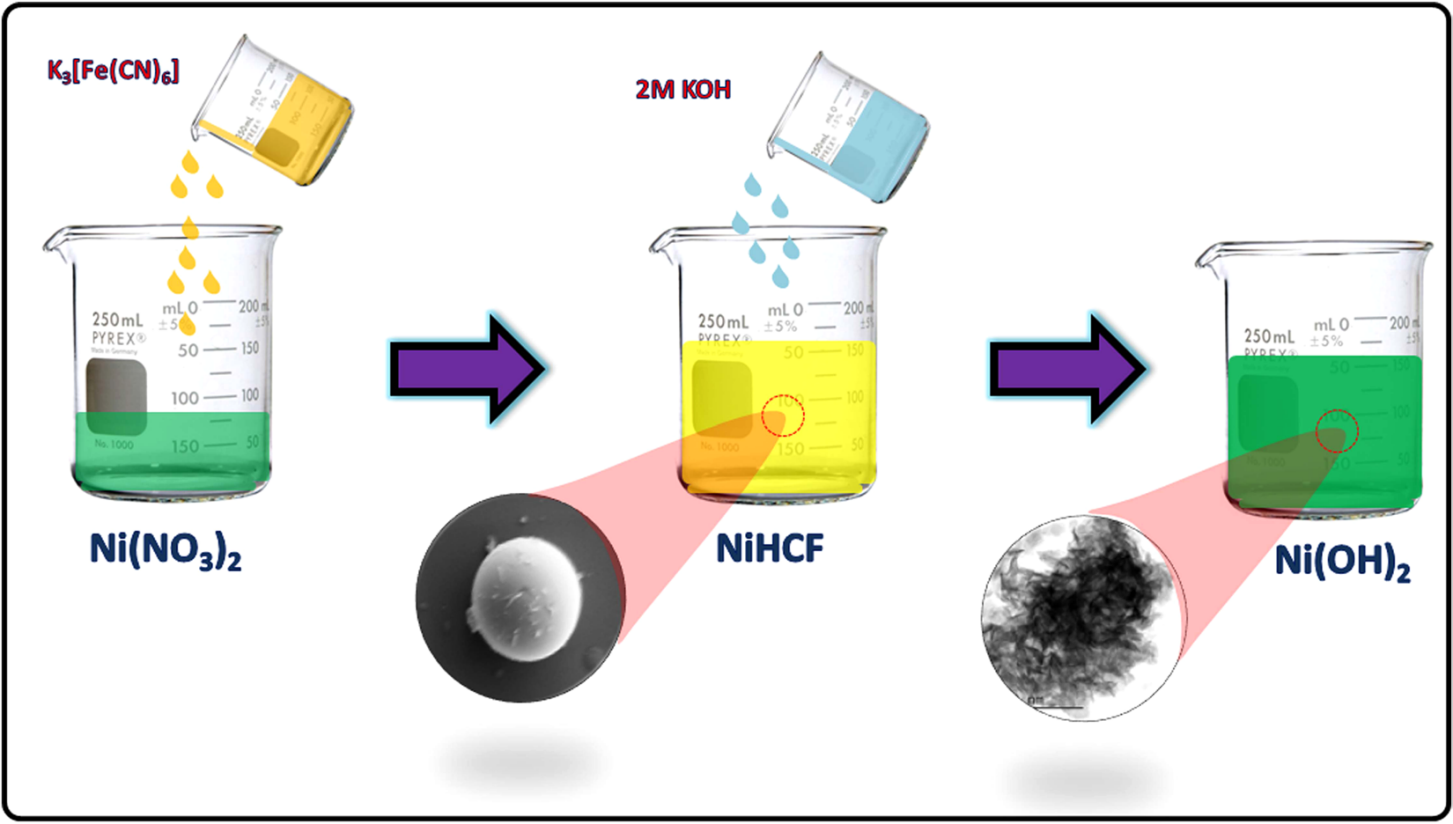
Schematic representation of Ni(OH)_2_ hexagonal nanosheets preparation *via* decomposition of NiHCF.

The schematic representation of Ni(OH)_2_ nanosheets synthesis *via* two step chemical route is shown in Figure 1. The first step is the formation of NiHCF complex from Ni(NO_3_)_2_ and K_3_[Fe(CN)_6_] and the second step concerned the chemical decomposition of NiHCF to Ni(OH)_2_ nanosheets. The overall chemical reactions involved in the formation of Ni(OH)_2_ nanosheets are shown in equations (1) and (2) as follows^20,21,45^:

1 $$Ni{(N{O}_{3})}_{2}.6\,{H}_{2}O+{K}_{3}[Fe{(CN)}_{6}]\to KNi[Fe{(CN)}_{6}]\downarrow +\,2\,KN{O}_{3}+6{H}_{2}O$$

2 $$KNi[Fe{(CN)}_{6}]+2\,KOH\to Ni{(OH)}_{2}\downarrow +3\,{K}^{+}+{[Fe{(CN)}_{6}]}^{3-}$$

**Figure 2:**
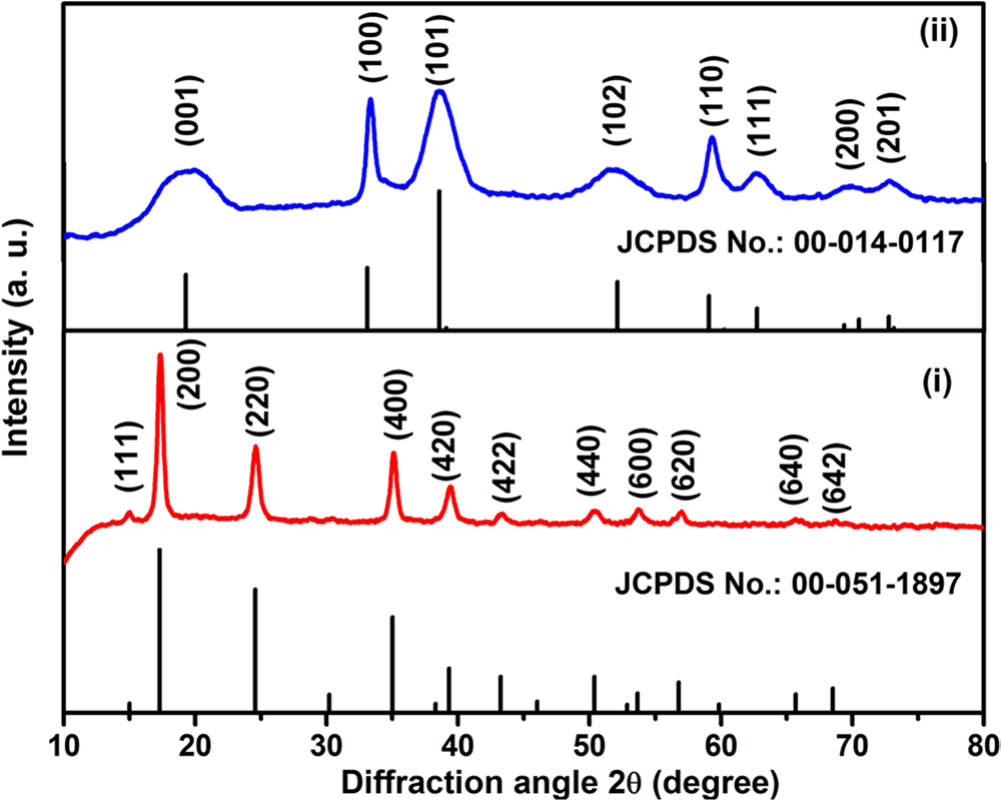
XRD pattern of (i) NiHCF and (ii) Ni(OH)_2_ nanosheets.

Powder X-ray diffraction is a fundamental analytical tool to examine the crystalline nature of the materials. The XRD profile of (i) NiHCF nanoparticles and (ii) Ni(OH)_2_ nanosheets are indexed with the standard ICDD Card No. 00-051-1897 (cubic, Fm-3m space group, a = 10.2340) and 00-014-0117 (hexagonal, P-3m1, a = b = 3.1260 and c = 4.6050), respectively (Fig. 2). The as-synthesized NiHCF shows a high intense peak which denotes the high crystallinity of the metal complex. The chemical decomposition of NiHCF results the formation of β-Ni(OH)_2_, which exhibits a set of diffraction peaks that are well indexed with the standard ICDD card no. 00-014-0117. The broad and high intense XRD profile indicates the formation of Ni(OH)_2_ nanosheets with ultra-small grain size and high crystalline nature, respectively. The characteristic peaks at 19.25°, 33.11°, 38.5°, 52.09°, 59.10°, 62.73°, 69.37° and 72.75° corresponding to (001), (100), (101), (102), (110), (111), (200) and (201) diffraction planes confirms the formation of crystalline Ni(OH)_2_, respectively. The crystallite size of Ni(OH)_2_ nanosheets was calculated by Debye–Scherrer^46^ equation and calculated crystallite size is ~10 nm. In addition, no other additional peaks were observed in the XRD pattern which confirms the phase pure formation of Ni(OH)_2_ nanosheets. It is noteworthy to mention here that the formation of Ni(OH)_2_ *via* chemical decomposition of NiHCF is simple and efficient root without any high temperature (or) pressure and it could be scalable for large scale synthesis of Ni(OH)_2_ nanosheets.

**Figure 3:**
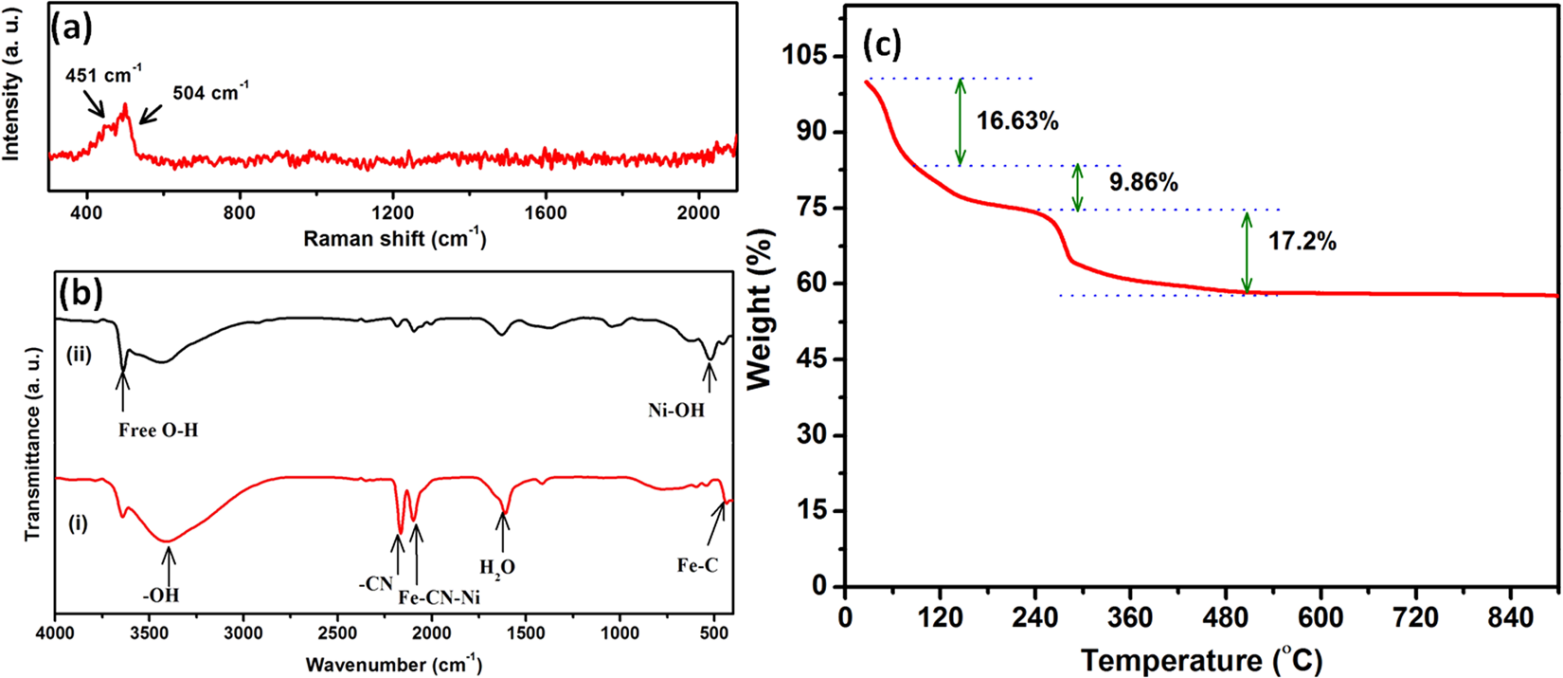
(**a**) Raman spectra of Ni(OH)_2_ nanosheets, (**b**) FT-IR spectra of (i) NiHCF and (ii) Ni(OH)_2_ nanosheets and (**c**) TGA profile of Ni(OH)_2_ nanosheets.

Figure 3a reveals that the Raman spectra of Ni(OH)_2_ which comprise of two peaks at 451 and 504 cm^−1^ attributed to the symmetric Ni-OH stretching mode and vibration of the Ni-O stretching mode and modes associated with the structural defects, respectively^47^. The FT-IR spectrum of NiHCF shows (Figure 3b**)** two major peaks at 2163 and 2099 cm^−1^ while are assigned for CN stretching vibration of -CN bound with metal ion within the lattice matrix viz. Fe^III^-CN-Ni^II^ and Fe^II^-CN-Ni^II^ environment, respectively^48^. Similarly, Ni(OH)_2_ nanosheets show (Figure 3b) four characteristic sharp bands at 518, 1626, 3412 and 3639 cm^−1^ due to Ni–OH stretching vibration, bending vibration mode of the water molecules, and the O–H stretching mode, which is characteristic of free O–H group of the brucite like structure^47,49^. This results confirm that the absence of interlayer water molecules and hence attributed to the flexing vibration of free hydroxyl existed in β - phase of Ni(OH)_2_.

Thermogravimetric analysis (TGA) indicates the change in the mass of the samples when the temperature increased as a function of time. This method is worthwhile for hydrated materials as the removal of water can be measured when the temperature of the sample is increased. TGA profile of Ni(OH)_2_ carried out in the temperature range of 30 to 900 °C with heating rate of 10 °C/min in air (Figure 3c**)**. From the TG profile, slight weight loss is observed in the temperature below 100 °C due to the removal of surface adsorbed water molecules. On further increasing the temperature, there is a significant weight loss (~9.86 wt. %) due to the removal of incorporated water between 100 to 160 °C. The sharp weight loss observed between 170 to 500 °C is attributed to the decomposition of Ni(OH)_2_ to NiO. The removal of the interlayer water may be occurred together with the decomposition of the Ni(OH)_2_ to NiO. The total weight loss measured was ∼17.2%, it is in good agreement with the theoretical value of 19.4% calculated from the following reaction^50^:

3 $$Ni{(OH)}_{2}\mathop{\to }\limits^{endothermic}NiO+{H}_{2}O$$

There is no further weight loss occurred at high temperature (500 to 900 °C), which confirms that all the Ni(OH)_2_ is converted to NiO.

**Figure 4:**
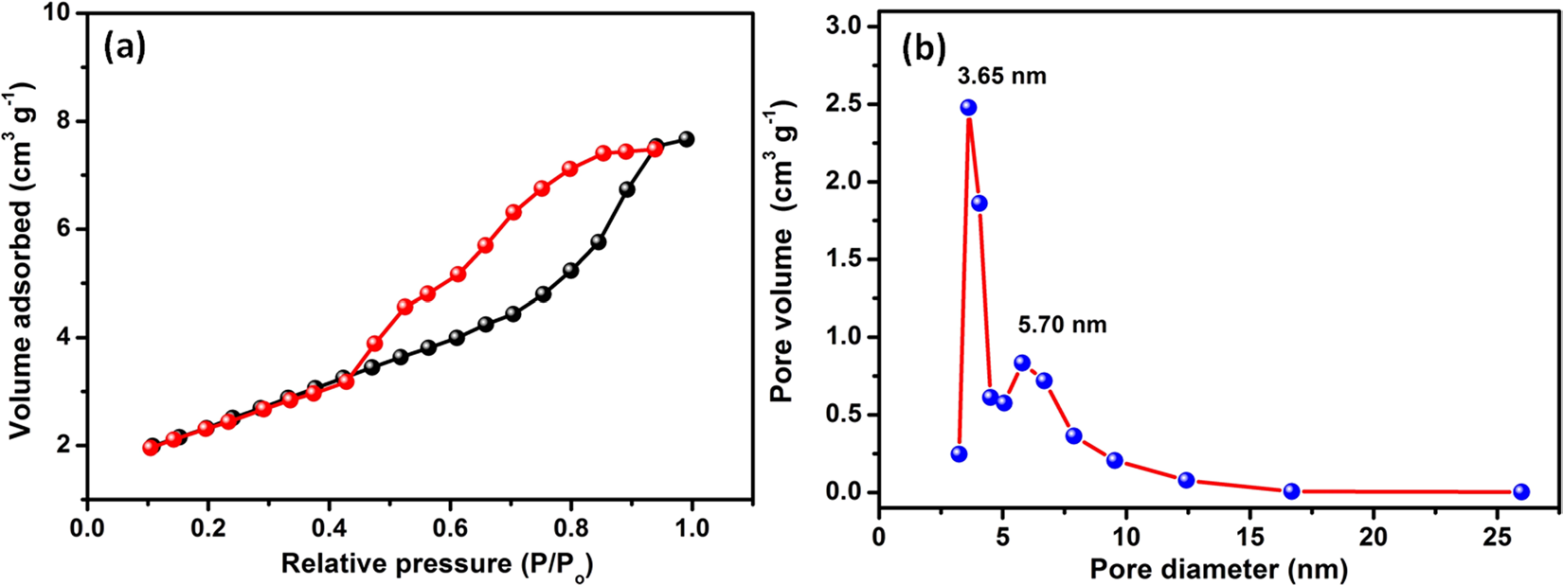
(**a**) BET nitrogen adsorption - desorption isotherm and (**b**) BJH pore size distribution curve of Ni(OH)_2_ nanosheets.

It is noteworthy here that, the decomposition of NiHCF complex by alkali results the formation of mesoporous Ni(OH)_2_ nanosheets. The N_2_ adsorption/desorption isotherm of Ni(OH)_2_ nanosheets shows a typical type-IV isotherm, according to the International Union of Pure and Applied Chemistry (IUPAC) classification, which is the characteristic of mesoporous materials (Fig. 4a). The BET specific surface area (SSA) and pore volume of Ni(OH)_2_ nanosheets is 206 m^2^/g and 0.270 cm^3^/g, respectively. The SSA of Ni(OH)_2_ nanosheets obtained in this synthesis method is higher than the previously reported method for the synthesis of Ni(OH)_2_ based electrode materials^51^. (See Supporting Information Table S1) The BJH pore size distribution study indicates that the synthesized Ni(OH)_2_ nanosheets has a pore diameter in the range of 3.6 to 5.7 nm (Fig. 4b). The observed high SSA and high pore volume will facilitate the diffusion of electrolytes through the pores to access the maximum surface and decrease the electron transport path, which is of huge benefits during the charge–discharge process in supercapacitors.

**Figure 5:**
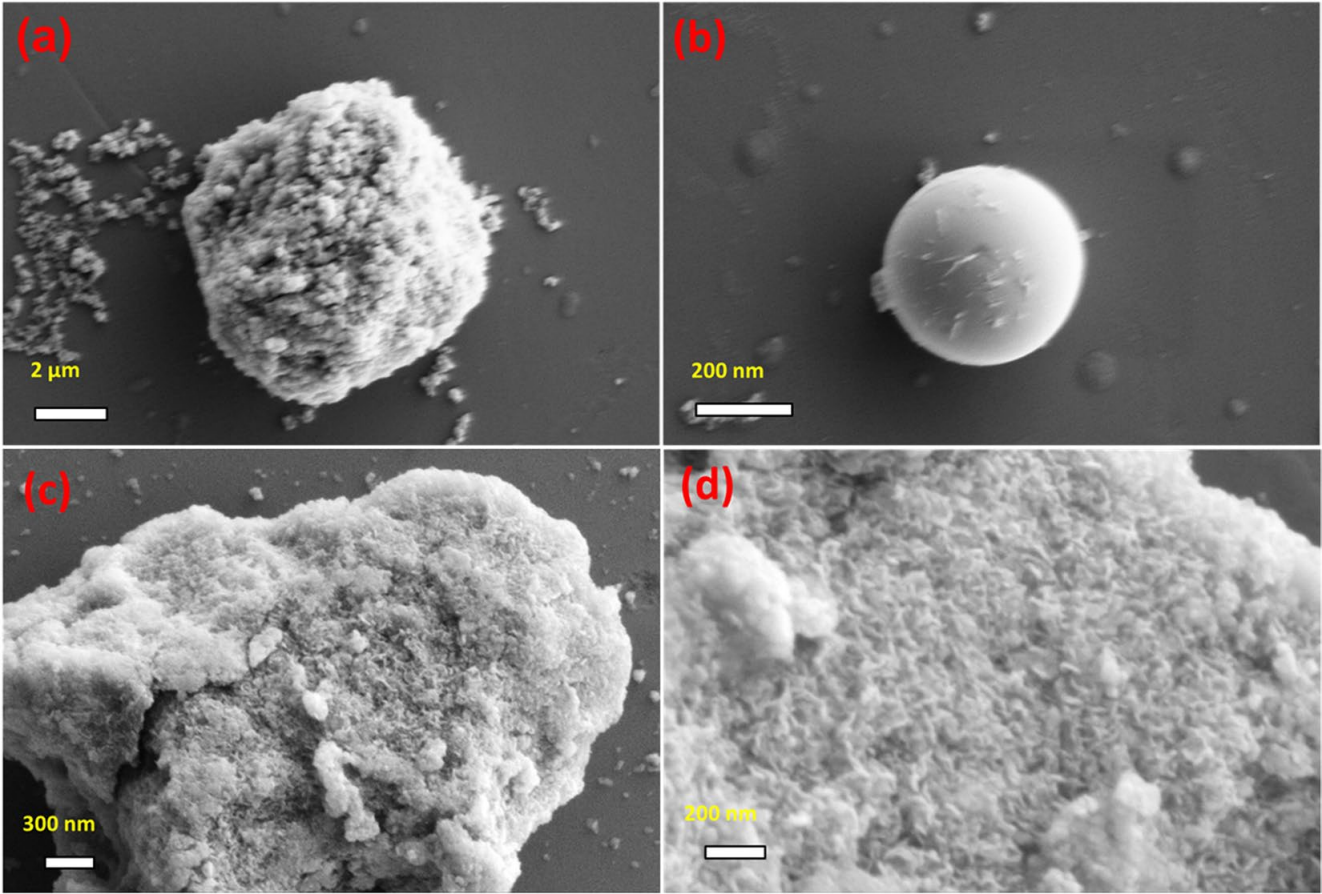
FE-SEM images of (**a**,**b**) NiHCF nanoparticles and (**c**,**d**) Ni(OH)_2_ nanosheets at different magnifications.

**Figure 6:**
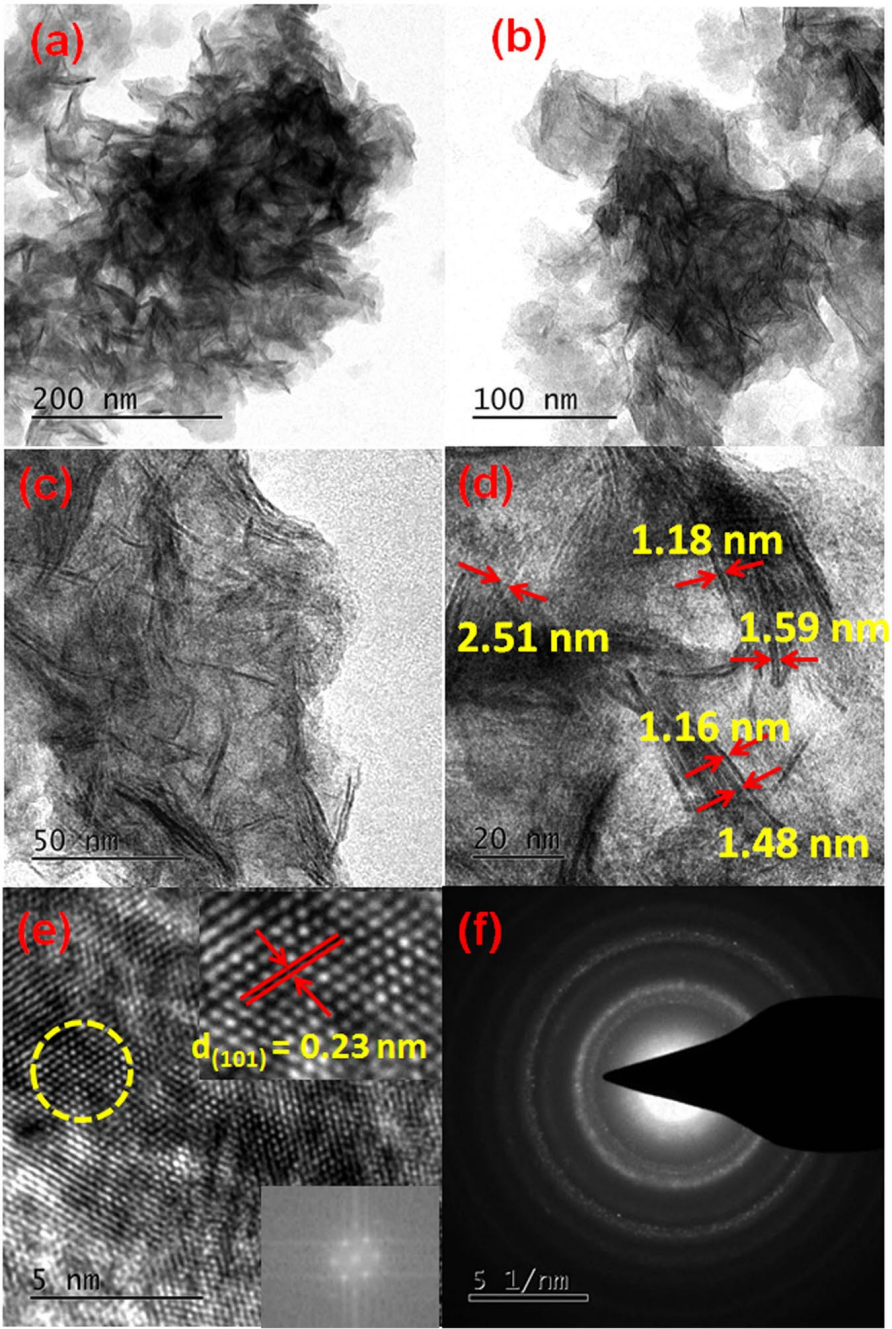
HR-TEM images of (**a**–**e**) Ni(OH)_2_ nanosheets at different magnifications and (**f**) SAED pattern of Ni(OH)_2_ nanosheets.

The surface morphology and particle size of synthesized materials were examined using FE-SEM analysis. Figure 5(a–d) represents the FE-SEM images of NiHCF and Ni(OH)_2_ nanosheets, respectively. When the sphere-like NiHCF nanoparticles decomposed by alkali treatment, a hierarchical sheet-like Ni(OH)_2_ are obtained. The spongy like hierarchical Ni(OH)_2_ nanosheets are further analyzed using HR-TEM analysis (Figure 6). From the images in Fig. 6(a–c), it is evident that Ni(OH)_2_ nanosheets consists of homogeneous dispersion of ultra-fine nanosheets which are hierarchically assembled. Higher magnification HR-TEM image of Ni(OH)_2_ nanosheets (Figure 6d**)** reveals that nanosheets thickness are in the range of 1~3 nm in size. The observed lattice fringe with *d* spacing of 0.23 nm corresponding to (101) plane of hexagonal Ni(OH)_2_ confirmed the formation of crystalline Ni(OH)_2_ nanosheets. (Figure 6e**)** The observed electron diffraction pattern further confirms the formation of poly crystalline Ni(OH)_2_ nanosheets. (Figure 6f) Based on the above electron microscopic observations, it is distinct that the spherical NiHCF nanoparticles are formed during the first step. Then, it decomposed by alkali treatment and results in the formation of Ni(OH)_2_ nanosheets with sheets thickness of 1–3 nm during the second step.

### Electrochemical analysis

#### Positive electrode materials

**Figure 7:**
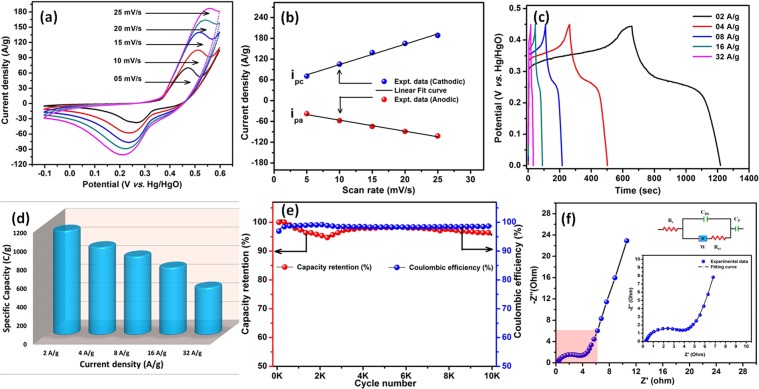
(**a**) CV profile of Ni(OH)_2_ nanosheets at different scan rate, (**b**) peak current density as a function of scan rate, (**c**) CD profile of Ni(OH)_2_ nanosheets at different current densities and (**d**) specific capacity as a function of current density, (**e**) capacity retention as a function of cycle number and (**f**) EIS Nyquist plots of Ni(OH)_2_ nanosheets.

Cyclic voltammetry (CV), galvanostatic charge-discharge (CD) and electrochemical impedance spectroscopy (ESI) analysis is a characteristics tool to evaluate the electrochemical performance of electrode materials. The as-synthesized Ni(OH)_2_ nanosheets for supercapacitor applications was evaluated using CV in the potential range of −0.1 to 0.6 V (*vs*. Hg/HgO) with various scan rate ranging from 5 to 25 mV/s (Figure 7a). The CV profile exhibits a strong redox peaks which is undoubtedly confirms the presence of faradaic behavior in Ni(OH)_2_ nanosheets. The oxidation and reduction peaks in the anodic and cathodic regions in the KOH electrolyte is attributed to the reversible faradaic process between Ni^2+^/Ni^3+^ anions, respectively. Based on the literature, the possible redox reactions can be described as follows^52,53^:

4 $$Ni{(OH)}_{2}+O{H}^{-}\rightleftharpoons NiOOH+{H}_{2}O+{e}^{-}$$

The observed redox peaks involve OH^−^ diffusion from the electrolyte to the electrode surface and the electrode to the solution during the reduction and oxidation process, respectively. The linear relationship of redox peak current and the scan rate proves that the electrode surface reaction at all scan rates is diffusion-controlled (Figure 7b**)**.

The specific capacity of the electrode material was calculated using CD experiment in the potential range of 0 to 0.45 V (*vs*. Hg/HgO) at various current densities (Figure 7c**)**. The observed non-linear discharge profile with humps in CD profiles at all current densities, confirms the faradaic influence of the electrode materials. The CD profile of Ni(OH)_2_ shows very high specific capacity of 1126, 948, 848, 728 and 512 C/g at the current densities of 2, 4, 8, 16, and 32 A/g, respectively. The studies on specific capacity as a function of current density shows that, 46% of initial specific capacity was retained at high current density of 32 A/g (Figure 7d**)**. The above observation demonstrates that the Ni(OH)_2_ electrode possess an excellent high rate capabilities for high power applications. The long-term electrochemical stability of Ni(OH)_2_ nanosheets electrode is tested for 10,000 charge-discharge cycles at high current density of 32 A/g **(**Figure 7e**)**. At the end of 10,000 cycling, a 96% of initial capacity is retained, which indicates the excellent electrochemical stability of the fabricated electrode materials. Moreover, the columbic efficiency of ~99% was retained for all 10,000 cycles, which specifies the high reversibility of electrode materials for long term performance in supercapacitors.

EIS is basic characteristic tools to understand the charge transfer process and the resistance associated with the charge storage at the electrode/electrolyte interface. The EIS of Ni(OH)_2_ nanosheets performed and the resulting Nyquist plot is shown in Figure 7f. The Nyquist plot was fitted with an appropriate equivalent circuit which containing various resistance and capacitance components. (Figure 7f (inset) The Nyquist plot contains the presence of solution resistance or bulk resistance (R_s_) which is associated with the resistance of electrolyte solution and other external resistance^38^. The semicircle at the high frequency region corresponds to the combination of charge-transfer resistance (R_ct_) and double layer capacitance (C_dl_) at the electrode/electrolyte interface^13^. Also, linear line at low frequency regions is greater than 45° towards to the imaginary axis is due to the electrolyte ions and proton diffusion on the electrode surface of active materials is denoted as Warburg impedance (W) or diffusion resistance^13^. The electrode material exhibited a very low R_s_ and R_ct_ values of 0.21 and 3.9 Ω, respectively. Thus clearly indicates that the less ionic resistance of the electrolyte with good electrical conductivity at the electrode/electrolyte interface. This may be ascribed to the hierarchical morphology of Ni(OH)_2_ nanosheets that assist for better electron transport during charge-discharge cycling. The faradaic component (C_F_) directs the electrochemical faradaic reaction of Ni(OH)_2_ nanosheets in KOH medium.

#### Negative electrode materials

**Figure 8:**
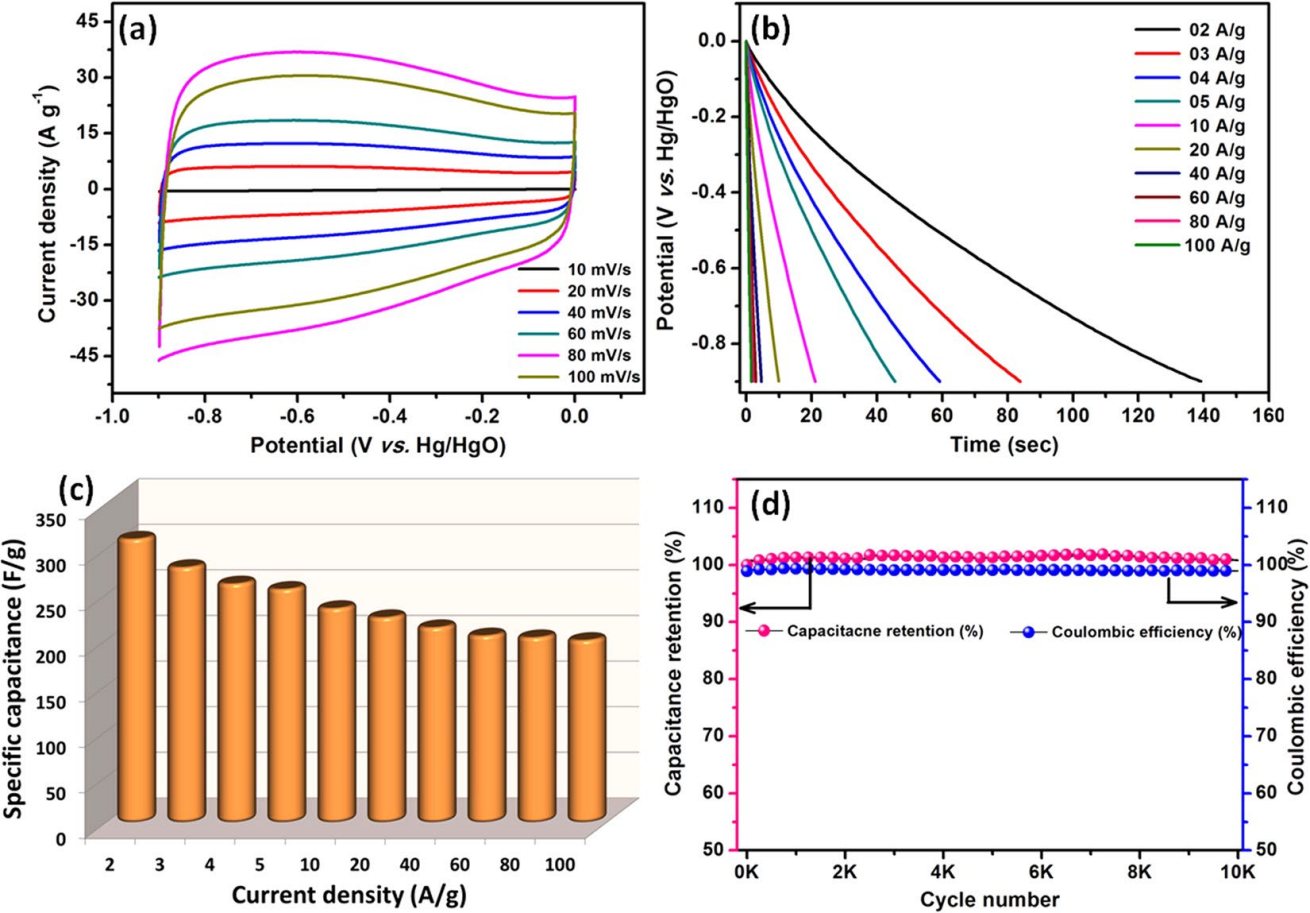
(**a**) CV and (**b**) discharge profile of OPAA-700 at different scan rate and current densities, respectively; (**c**) specific capacitance as a function of current density and (**d**) capacitance retention and coulombic efficiency of OPAA-700 electrode as a function of cycle numbers.

In our earlier study, we discussed that the orange peel derived activated carbon (OPAA-700) can be used as an negative electrodes material for full cell fabrications^38^. The electrochemical performance of OPAA-700 in 3.5 M KOH electrolyte is studied using CV and CD analysis in the potential range of −0.9 to 0 V (*vs*. Hg/HgO) at various scan rates and current densities, respectively. The CV profile of OPAA-700 shows an ideal rectangular shape with mirror image characteristics of EDLCs contribution **(**Figure 8a**)**. The discharge profile of OPAA-700 at various current densities ranging from 2 to 100 A/g, clearly exhibits a linear line due to the perfect EDLC capacitive behavior of the OPAA-700 electrode materials **(**Figure 8b**)**. The OPAA-700 electrode shows a specific capacitance of 311 F/g at current density of 2 A/g. When the current density is increased to 100 A/g, the specific capacitance values still retained as 200 F/g which implies that the electrode has a good rate performance. Figure 8c reveals the specific capacitance as a function of different current density, even at high current density of 100 A/g, the OPAA-700 showed 64% of initial specific capacitance which specify the excellent rate performance. The 3-D interconnected porous network and high electrical conductivity of OPAA-700 facilitates good accessibility of electrode surface at high current density and to enhance the electron charge transfer reaction at the interface for fast surface adsorption - desorption reaction, respectively.

The long-term stability of the OPAA-700 was studied by cycling for 10,000 charge-discharges at high current density of 100 A/g. (Figure 8d**)** After cycling, 100% of initial capacitance is retained, which ensures the excellent electrochemical stability of the fabricated electrode materials. Moreover, the observed high columbic efficiency of ~99.5% for all 10,000 cycles confirmed the high electrochemical stability of the electrode materials for high rate performance. The observed high specific capacitance and excellent electrochemical stability facilitates OPAA-700 as negative electrode materials for further asymmetric supercapacitors fabrications.

#### Non-aqueous symmetric supercapacitor

**Figure 9:**
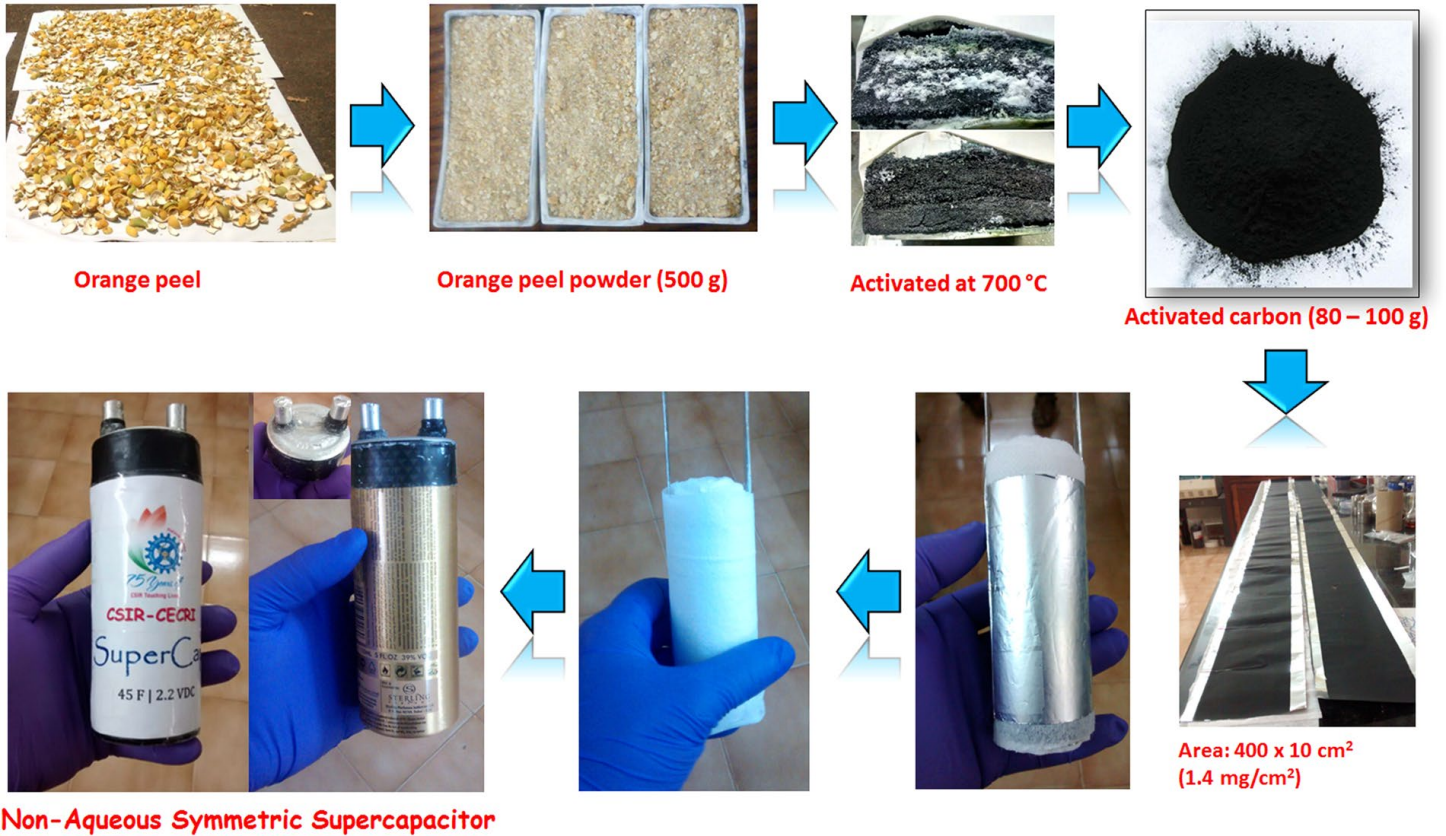
A step-by-step fabrications of non-aqueous symmetric supercapacitor device.

Generally, activated carbon electrode materials are extensively used as electrode materials for non-aqueous supercapacitor with an expensive ionic liquid and organic electrolytes. In order to evaluate our activated carbon materials similar to commercial non-aqueous symmetric supercapacitor, a large size device is fabricated. The synthesize of OPAA-700 electrode materials are optimized to 80 to 100 g per batch and the electrode material slurry was prepared using ball-milling (400 rpm for 6 h). The electrode slurry was coated over the Al foil substrate (400 × 10 cm^2^) with a mass loading of 1.4 mg/cm^2^ as shown in Figure 9. A cylindrical type cell was fabricated by rolling two electrodes with nonwoven foam as separator. This rolled electrode materials are placed in Al container and filled with tetraethylammonium tetrafluoroborate (TEABF_4_) in acetonitrile. The entire device fabrication was carried out inside the Ar filled glove box (GS MEGA E-Line, Germany).

**Figure 10:**
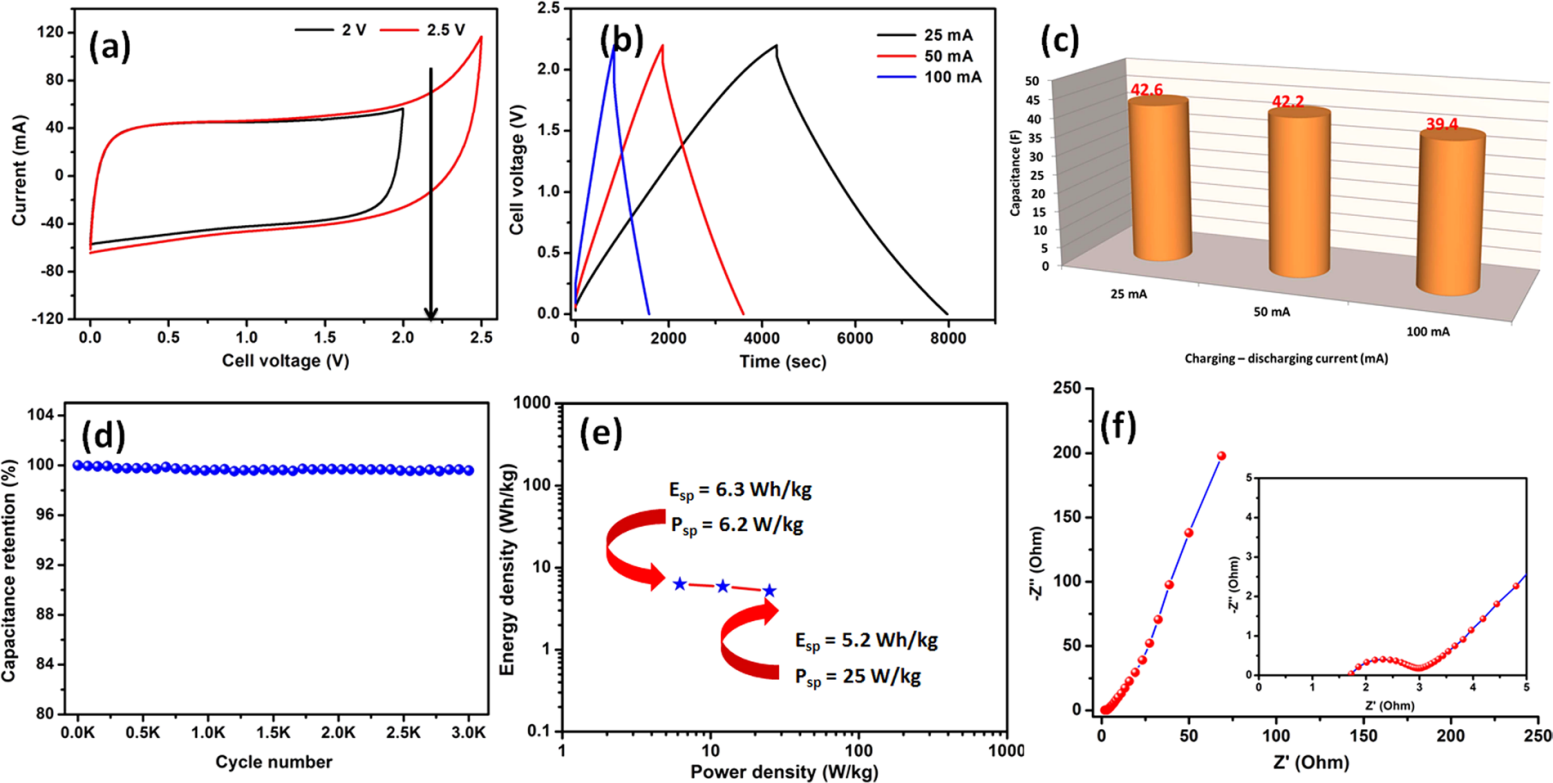
(**a**) CV profile of fabricated symmetric supercapacitor in non-aqueous electrolyte with different cell voltage; (b) CD profile of fabricated symmetric supercapacitor cell voltage of 2.2 V with different charging – discharging current; (**c**) capacitance as a function of discharging current, (**d**) capacitance retention as a function of cycle numbers, (**e**) Ragone plot for fabricated symmetric supercapacitor and (f) typical Nyquist plot for fabricated symmetric supercapacitors.

Figure 10a reveals the CV profile of fabricated symmetric supercapacitor in non-aqueous electrolyte with different cell voltage. From the ideal rectangular shape profile, the EDLC behaviour of the fabricated device could be confirmed up to cell voltage of 2.2 V. The CD profile of fabricated device with different charging-discharging current is shown in Figure 10b. From the discharge profile, the calculated actual stored capacitance (C_st_) values of 42.6, 42.2 and 39.4 F at charging-discharging current of 25, 50 and 100 mA, respectively. In terms of specific capacitance (C_sp_), the fabricated device delivers a high specific capacitance of 10.2, 10.1 and 9.4 F/g at a charging-discharging current of 25, 50 and 100 mA, respectively. Even at higher current, the device retains their capacitance values more than 92% which indicates the good rate performance of fabricated symmetric supercapacitors. (Figure 10c**)** The long-term stability of the fabricated symmetric supercapacitor was studied by cycling for 3,000 charge-discharges at high charging-discharging current of 100 mA. (Figure 10d**)** After cycling, 98% of initial capacitance is retained, which ensures the excellent electrochemical stability of the fabricated symmetric supercapacitor.

Typical Ragone plot for the fabricated symmetric supercapacitor device is shown in Fig. 10e. The fabricated device delivered a specific energy density (E_sp_) and power density (P_ps_) of 6.3 Wh/kg and 6.2 W/kg at 25 mA charging current, respectively. When the charging current increased to 100 mA, the fabricated symmetric supercapacitor device delivered an E_st_ and P_st_ of 5.2 Wh/kg and 25 W/kg, respectively. It is worthy to note here that the actual E_st_ and P_st_ of the fabricated symmetric supercapacitor device is 26.9 mWh and 27.3 mW at 25 mA charging current, respectively. When the charging current increased to 100 mA, the fabricated symmetric supercapacitor device delivered an E_st_ and P_st_ of 19.8 mWh and 95 mW, respectively. Figure 10f reveals the typical Nyquist plot of fabricated symmetric supercapacitor device which exhibit R_s_ and R_ct_ value of ~1.6 and 1.3 Ω, respectively. The EIS studies confirm that the fabricated symmetric supercapacitor device with non-aqueous electrolyte holds an excellent electrical contact with minimum ionic resistance suitable for viable applications.

#### Aqueous asymmetric supercapacitor

Generally, activated carbon electrode materials are extensively used as electrode materials for non-aqueous supercapacitor with expensive ionic liquid and organic electrolytes. Even though supercapacitors has an significant advantages such as high power, long cycle life, wider operating temperature, less maintenance and eco-friendly in nature, these electrolyte reduces energy density and adds high cost that limits supercapacitor as alternative for conventional lead-acid and lithium-ion batteries^2,54^. The cost of the non-aqueous based supercapacitors device 2400$/KWh is significantly higher than the convention batteries^55^. Supercapacitors in aqueous electrolytes will be inexpensive, because of the replacement of expensive ionic/orgonic electrolytes and simplicity in electrode materials processing^55,56^. According to the literature, material costs found to be approx. 70% of overall costs, leaving the remaining 30% to the production^55^. Among the 70% of materials cost, it was found that electrolyte (conductive salt and solvent) and NMP solvent used for the making the electrode paste contributions is major^55^. In order to minimize the materials cost, especially for electrolyte, aqueous based electrolytes could be used. But the low cell voltage of symmetric aqueous based supercapacitor restricts their potential application due to water decomposition. Recently, asymmetric hybrid supercapacitors in aqueous electrolytes are developed in which the faradaic metal oxide/hydroxide/sulfide and an EDLC based carbon electrode materials are used as positive and negative electrodes, respectively^15,57^. The advantage of this hybrid asymmetric supercapacitor is (i) higher cell voltage can be possible in aqueous electrolyte and (ii) higher energy density. In asymmetric configurations, both non-faradaic and faradaic processes are used to store charges in a single cell^58–60^.

**Figure 11:**
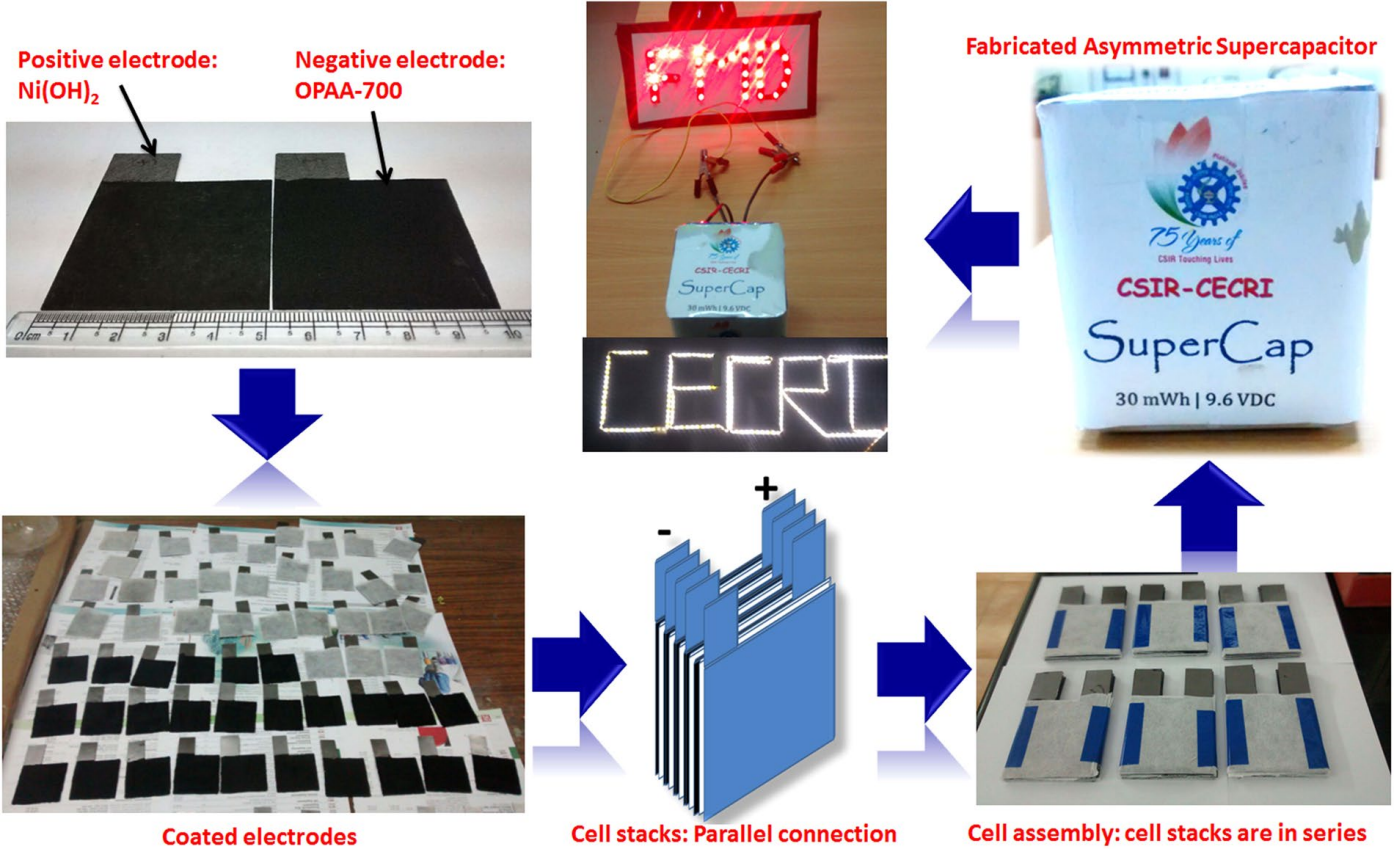
A step-by-step fabrication of asymmetric supercapacitor device.

In our study, the large scale fabrication of asymmetric supercapacitor was demonstrated using NiHCF derived Ni(OH)_2_ nanosheets and OPAA-700 electrode materials as positive and negative electrodes, respectively. The electrode paste were prepared similar to the half-cell measurements (three electrode configurations) described earlier. It is important to mention, the amount of charge stored in negative electrodes and positive should be equal for better performance. It depends on the specific capacitance or capacity and working potential of electrode materials at constant current density^61–63^. Based on the three electrode studies, the calculated optimal mass ratio of positive electrode (m_+_) and negative electrode (m_-_) is 0.25 for fabrications of asymmetric supercapacitors. Thus, charge balance was carried out by loading appropriate amount of active materials. The cell voltage of fabricated asymmetric supercapacitors (5 × 5 cm^2^) in aqueous electrolyte is 1.6 V. The mass loading on negative electrodes side is ~8 mg/cm^2^ and positive side is ~2 mg/cm^2^. In a single cell stack contains six cells in parallel connection using back to back coating (end plates are single side and other plated are double side coated). And, the full cell assembly was made *via* six cell stacks that are connected in series to make an assembly of asymmetric supercapacitors device. (Fig. 11).

**Figure 12:**
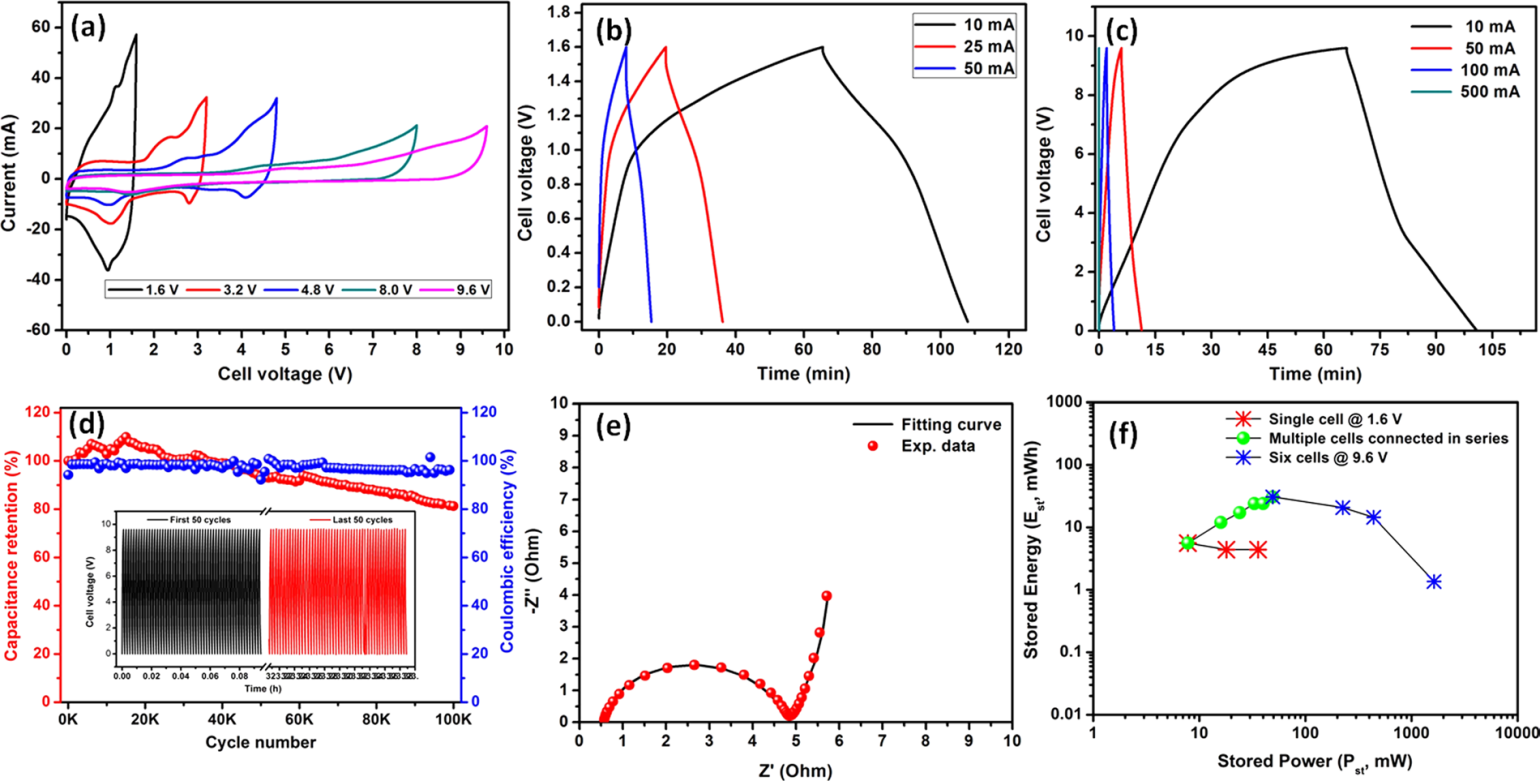
(**a**) CV profile of fabricated asymmetric supercapacitor with different number of cells stacks connected in series; CD profile of fabricated asymmetric supercapacitor with (**b**) 1.6 V and (**c**) 9.6 V at different charging-discharging current; (**d**) capacitance retention and coulombic efficiency as a function of cycle numbers and first and last 50 cycles (inset), (**e**) typical Nyquist plot for fabricated asymmetric supercapacitors and (**f**) Ragone plot for fabricated asymmetric supercapacitor with single cell and serially connected six cells.

Electrochemical performance of the fabricated asymmetric supercapacitor cells were evaluated using CV, CD and EIS analysis. Figure 12a shows the CV profile of asymmetric supercapacitor with single, two, three, five and six cells connected in series at scan rate of 1 mV/s. Even at cell voltage of 9.6 V, the assembly is stable with obvious redox peaks, and it shows a synergistic effect of EDLCs and faradaic capacitance in the device. Figure 12(b,c) reveals the CD profile of cells with a cell voltage of 1.6 V and 9.6 V at different charge-discharging current, respectively. A maximum C_t_ of 16 F (C_sp_ = 21.5 F/g) at 10 mA charging current was obtained with the cell voltage of 1.6 V. Further, when the cell voltage increased to 9.6 V by six cells connecting with step-by-step in series, the C_t_ decreased to 8.4, 5.4, 4.2, 2.9, 2.3 F for two, three, four, five and six cells at a constant charging current of 10 mA, respectively.

Long-term electrochemical stability of fabricated asymmetric supercapacitor device is essential for the viable applications. Thus, electrochemical stability of the fabricated asymmetric supercapacitor device was investigated by continuous cycling for 1,00,000 cycles with high charging current of 500 mA at the cell voltage of 9.6 V. Figure 12d shows the capacitance retention and coulombic efficiency of fabricated asymmetric supercapacitor device as a function of cycle number. From the graph, it is clear that more than 81% of capacitance was retained and maintain a constant coulombic efficiency of ~98.5% for all cycles, which shows an excellent electrochemical stability of fabricated asymmetric supercapacitor device at high cell voltage. First and last 50 continuous charge-discharge profiles are shown in Fig. 12d (inset). Figure 12e reveals the typical Nyquist plot of fabricated asymmetric supercapacitor device which exhibit R_s_ and R_ct_ value of ~0.5 and 4.5 Ω, respectively. From the EIS studies, it confirms the fabricated asymmetric supercapacitor device have an excellent electrical contact with minimum ionic resistance.

The real applications of supercapacitors were decided based on the E_st_ and P_st_ of the device. The typical Ragone plot of the fabricated asymmetric supercapacitor device with in the cell voltage of 1.6 V and 9.6 V are shown in Fig. 12(f). The single cell stack delivered a high E_st_ and P_st_ of 5.7 mWh and 8 mW at 10 mA charging current, respectively. When charging current is increased to 50 mA, the device delivered high P_st_ of 36 mW with a E_st_ of 4.4 mWh. The fabricated device with two, three, four, five and six cells assembly delivered an E_st_ of 12, 17.3, 24, 2.8 and 30 mWh, respectively. Similarly, device with two, three, four, five and six cells stacked assembly delivered a P_st_ of 16, 24, 33, 43 and 50 mW, respectively. Connecting the cells in series results the increase in the cell voltage with decreasing capacitance of the device. The six cells assembly connected in in series delivered a E_st_ and P_st_ of 30 mWh and 50 mW at 10 mA charging current, respectively. When the charge current increased to 500 mA, the fabricated asymmetric supercapacitor device delivered a E_st_ and P_st_ of 2.5 mWh and 1632 mW, respectively. For comparison with the reported works, the E_sp_ and P_sp_ of the fabricated asymmetric supercapacitor device was calculated. The single cell stack delivered a high E_sp_ and P_sp_ of 7.7 Wh/kg and 10.8 W/kg at 10 mA charging current, respectively. (Figure S1) The fabricated device with two, three, four, five and six cells stacked assembly delivered an E_sp_ of 8, 7.7, 6.8, 6.8 and 6.4 Wh/kg, respectively. Similarly, device with two, three, four, five and six cells stacked assembly delivered a P_sp_ of 10.7, 10.7, 9.1, 11.4, and 10.4 W/kg, respectively. The detailed values are shown in Tables S2, S3 and S4. To demonstrate the potential application of the fabricated asymmetric supercapacitor device, a white light emitting diode (LED) was connected to the device (LEDs strips containing 285 white LEDs) and it could light up for more than 3 minutes (SV1, Supplementary Information). From the above observation, the E_sp_, E_st_, P_sp_ and P_st_ of the fabricated asymmetric supercapacitor device is still comparable to the commercially available non-aqueous based supercapacitor with equal mass loading.

## Conclusions

Hexagonal Ni(OH)_2_ nanosheets preparation via a two-step method using chemical decomposition of NiHCF was demonstrated. The FE-SEM and HR-TEM analysis of Ni(OH)_2_ nanosheets revealed the formation of hierarchical nanosheets that are randomly oriented. The nitrogen adsorption - desorption isotherms of Ni(OH)_2_ nanosheets showed the high specific surface area and pore volume of 206 m^2^/g and 0.270 cm^3^/g with mesoporous nature, respectively. A high specific capacity of 1126 C/g at current density of 2 A/g with good rate performance and excellent electrochemical stability (~96%) was achieved. Activated carbon was prepared from orange peel waste *via* a carbonization followed by chemical activation. Asymmetric supercapacitors were fabricated in aqueous electrolyte to exhibit a stable device voltage of 1.6 V for single cell that delivered a high E_st_ and P_st_ of 5.7 mWh and 36 mW, respectively. Also, asymmetric supercapacitor stack with six cells connected in series assembly delivered 9.6 V, high E_st_ and P_st_ of 30 mWh and 1632 mW, respectively. Even 81% of initial capacitance retained after 1,00,000 charge-discharges cycling at high charging current of 500 mA which shows an excellent electrochemical stability of fabricated asymmetric supercapacitors for viable applications.

## Methods

### Materials

Nickel nitrate (Ni(NO_3_)_2_. 6H_2_O, 99%), potassium ferricyanide (K_3_[Fe(CN)_6_], 99 wt. %), polyvinylidene fluoride (PVDF) and potassium hydroxide pellets (KOH, 98 wt. %) were purchased from Sigma-Aldrich, India. Ethanol (CH_3_CH_2_OH, 99 wt. %) was purchased from SRL Pvt. Ltd, India. All purchased chemicals and reagents were in analytical grade and used as received without any further purification. Deionized (DI) water was obtained through MILLIPORE water system.

### Synthesis of Ni(OH)_2_ nanosheets

Ni(OH)_2_ nanosheets was prepared by two step chemical route *via* formation of nickel hexacyanoferrate (NiHCF) complex and chemical decomposition of NiHCF complex. (Fig. 1) In a typical synthesis process 10 mM Ni(NO_3_)_2_. 6H_2_O solution was slowly added to 10 mM of K_3_[Fe(CN)_6_], and the resulted mixture was stirred for 3 h. The obtained precipitate NiHCF was washed with DI water for many times until other impurities are completely removed and dried at 60 °C in a vacuum oven. The NiHCF powder was re-dispersed in DI water and one freshly prepared 2M KOH solution was added with continuous stirring. The resulting precipitate was filtered by nylon membrane (20 µm) vacuum filter and washed with DI water followed by ethanol until the filtrate became neutral. The obtained powder was dried at 60 °C in a vacuum oven and grained well to make a fine powder.

### Synthesis of bio-derived activated nanoporous carbon

In our study, orange peel derived activated nanoporous carbon (OPAA-700) activated at 700 °C was used as a negative electrodes material for full cell fabrications. The detailed synthesis procedure and characterizations are published in our previous publications^38^. In detail, the powdered orange peel sample was subjected to carbonization at 600 °C for 3 h in a tubular furnace under argon (Ar) atmosphere. For the chemical activation, the carbonized sample was mixed with KOH in a weight ratio of 1:3 and the obtained slurry was again heat-treated at 700 °C for 1 h in a tubular furnace under Ar atmosphere at a heating rate of 5 °C/min to obtain chemically activated carbon. The resulted activated carbon was washed thoroughly with 1 M HCl solution followed by DI water until the pH of the sample becomes neutral. The resulted activated carbon was dried at 80 °C for 5 h and denoted as orange-peel carbon after activation (OPAA-700).

### Materials characterization

The phase formation of the Ni(OH)_2_ nanosheets was examined by powder X-ray diffraction (XRD) measurements using a BRUKER D8 ADVANCE X-Ray Diffractometer with Cu K_α_ radiation (λ = 1.5418 Å) from 10° to 80° at 0.021 step size and a count time of 0.2 s. The chemical nature of Ni(OH)_2_ nanosheets was studied using a laser Raman spectroscopy (RENISHAW Ivia laser Raman microscope) outfitted with a semiconducting laser (wavelength λ = 633 nm). Fourier transform infrared (FT-IR) spectra were recorded on a TENSOR 27 spectrometer (Bruker) using the KBr pellet technique from 400 to 4000 cm^−1^. The morphology Ni(OH)_2_ nanosheets was characterized by field-emission scanning electron microscopy (FE-SEM, Carl Zeiss AG Supra 55VP) with an acceleration voltage of 5–30 kV. The particle size of Ni(OH)_2_ nanosheets was visualized using a higher resolution - transmission electron microscope (HR-TEM, TF20: Tecnai G^2^ 200 kV (FEI) with field emission gun (FEG)) working at an accelerating voltage of 200 kV. The thermal stability, chemical phase and composition of the synthesized Ni(OH)_2_ nanosheets was carried out to using Thermogravimetric analyses (TGA) with TGA/DTA instruments (Model SDT Q 600) from room temperature to 800 °C at a heating rate of 10 °C/min in air. BET- Nitrogen adsorption-desorption isotherm and pore-size distribution of the Ni(OH)_2_ nanosheets was measured using Quantachrome NOVA 3200e surface area and pore size analyzer.

### Electrochemical characterization

The working electrode was fabricated by mixing the active materials Ni(OH)_2_ nanosheets (or) bio-derived activated carbon, super-P carbon and polyvinylidene fluoride (PVDF) binder with a weight ratio of 75: 20: 5, respectively. This electrode material mixer was dispersed in N-Methyl-2-pyrrolidone (NMP) solvent to make slurry and this slurry was coated on flexible graphite paper substrate. The coated flexible graphite paper was dried at 100 °C for overnight and the loading of the electrode materials was ∼2 mg/cm^2^. The Ni(OH)_2_ nanosheets (or) bio-derived activated carbon coated graphite paper, Hg/HgO (20% KOH) and a slice of Pt foil were used as a working electrode, reference electrode and a counter-electrode, respectively. The electrochemical behaviour of electrode materials in 3.5M KOH (20% KOH) as electrolyte was characterized by CV, CD and ESI measurements. The CV and CD experiments were carried out at different scan rates and various current densities, respectively. The specific capacitance, energy density and power density of the electrodes were calculated from the discharge profile using the following formula^7,13,18^:

5 $${C}_{sp}=\frac{I{\rm{\Delta }}t}{m{\rm{\Delta }}V}\,$$

6 $$E=1/2C{V}^{2}$$

7 $$P=\frac{E}{{\rm{\Delta }}t}$$

Here, *C*_*sp*_ is the specific capacitance (F/g), *I* is the charge-discharge current (A), Δt is the total discharge time (sec), *m* is the total active mass of the electrode materials (g) and Δ*V* is potential window during discharge process (V). EIS experiments were performed with 0 V bias and a sinusoidal signal of 5 mV in a frequency range of 10 mHz to 100 kHz. EIS data were analyzed using a Nyquist plot.

## Supplementary information

Supplementary Information

Supplementary Information SV1

## Data Availability

Readers can access the data *via* contact to the authors.
