# Supplementary material for: Fabrication of 9.6 V High-performance Asymmetric Supercapacitors Stack Based on Nickel Hexacyanoferrate-derived Ni(OH)2 Nanosheets and Bio-derived Activated Carbon
Source: Sci Rep. 2019 Jan 31;9:1104. doi: 10.1038/s41598-018-37566-8 (PMC6355786; doi:10.1038/s41598-018-37566-8)
Supplement: Supplementary file 1 — Supplementary Information [file 41598_2018_37566_MOESM1_ESM.pdf]

Supplementary Information

Fabrication of 9.6 V High-performance Asymmetric Supercapacitors Stack Based on Nickel Hexacyanoferrate-derived $Ni(OH)_2$ Nanosheets and Bio-derived Activated Carbon

K. Subramani,[a,b] and M. Sathish[a,b]*

[a]Functional Materials Division, [b]Academy of Scientific and Innovative Research (AcSIR),
CSIR-Central Electrochemical Research Institute, Karaikudi – 630 003,
Tamil Nadu, India.

Corresponding authors: *marappan.sathish@gmail.com; msathish@cecri.res.in*

Supplementary Figures:

Table S1 Comparison of $Ni(OH)_2$ based electrode materials based on the surface morphology and surface area of the electrode materials for supercapacitor applications.

S. No.	Electrode materials	Surface morphology of $Ni(OH)_2$	Specific surface area (m^2/g)	Reference
1	$RGO/Ni(OH)_2$	Nanoplates	197	[1]
2	$CNT/Ni(OH)_2$	Core–shell	134.7	[2]
3	$CNF/MnO_2/Ni(OH)_2$	Nanosheets	262.8	[3]
4	$Ni(OH)_2/AC$	Nanoflakes	1086	[4]
5	$Ni(OH)_2$	Nanoflakes	11	[5]
6	$Ni(OH)_2$	Hollow spheres	93.2	[5]
7	$Ni(OH)_2/MWCNT$	Nanoparticles	153	[6]
8	$Ni(OH)_2$ $/MnO_2$	Nanowire	128.7	[7]
9	$Ni(OH)_2$	Nanoflakes	51.3	[8]
10	$NiO/Ni(OH)_2$	Nanoflakes	220	[9]
11	$CQDs/Ni(OH)_2$	Nanosheets	162.9	[10]
12	**$Ni(OH)_2$**	**Nanosheets**	**206**	**This work**

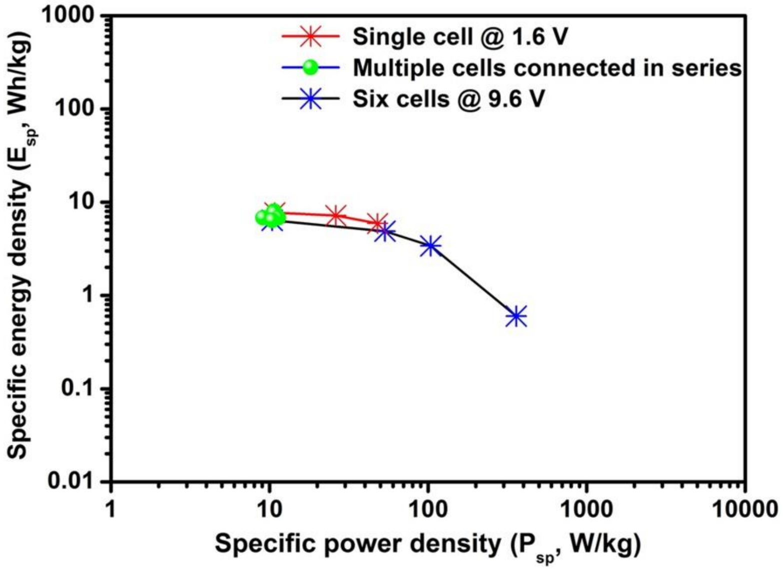

Figure S1 Ragone plot for fabricated asymmetric supercapacitor with single cell and serially connected six cells.

Table S2 Comparison of E_{sp}, P_{sp}, E_{st} and P_{st} of fabricated single stack asymmetric supercapacitor at different charging-discharging current.

Charging-discharging current (mA)	Actual storage		Specific storage	
	E_{st} (mWh)	P_{st} (mW)	E_{sp} (Wh/kg)	P_{sp} (W/kg)
10	5.7	8	7.7	10.8
25	5.4	19.5	7.2	26.2
50	4.4	36	5.9	48

Table S3 Comparison of E_{sp}, P_{sp}, E_{st} and P_{st} of fabricated asymmetric supercapacitor with multiple cell stacks are connected in series at a constant charging-discharging current of 10 mA.

Cell voltage (V)	Actual storage		Specific storage	
	E_{st} (mWh)	P_{st} (mW)	E_{sp} (Wh/kg)	P_{sp} (W/kg)
1.6	5.7	8	7.7	10.8
3.2	12	16	8	10.7
4.8	17.3	24	7.7	10.7
6.4	24	33	6.8	9.1
8.0	25.8	43	6.8	11.4
9.6	30	50	6.4	10.4

Table S4 Comparison of E_{sp}, P_{sp}, E_{st} and P_{st} of fabricated asymmetric supercapacitor assembly (9.6 V) at different charging-discharging current.

Charging-discharging current (mA)	Actual storage		Specific storage	
	E_{st} (mWh)	P_{st} (mW)	E_{sp} (Wh/kg)	P_{sp} (W/kg)
10	30	50	6.4	10.4
50	21.7	238	4.9	53.4
100	14.7	448	3.4	104
500	2.5	1632	0.6	360

References:

1. Zhang, C., Chen, Q. & Zhan, H. Supercapacitors Based on Reduced Graphene Oxide Nanofibers Supported $Ni(OH)_2$ Nanoplates with Enhanced Electrochemical Performance. *ACS Appl. Mater. Interfaces* **8,** 22977–22987 (2016).
2. Yi, H. *et al.* Advanced asymmetric supercapacitors based on CNT@$Ni(OH)_2$ core–shell composites and 3D graphene networks. *J. Mater. Chem. A* **3,** 19545–19555 (2015).
3. Zhou, D. *et al.* 3D interconnected networks of a ternary hierarchical carbon nanofiber/MnO_2/$Ni(OH)_2$ architecture as integrated electrodes for all-solid-state supercapacitors. *RSC Adv.* **6,** 71882–71892 (2016).
4. Gong, S., Cao, Q., Jin, L., Zhong, C. & Zhang, X. Electrodeposition of three-dimensional $Ni(OH)_2$ nanoflakes on partially crystallized activated carbon for high-performance supercapacitors. *J. Solid State Electrochem.* **20,** 619–628 (2016).
5. Tong, G.-X. *et al.* Polymorphous α- and β-$Ni(OH)_2$ complex architectures: morphological and phasal evolution mechanisms and enhanced catalytic activity as non-enzymatic glucose sensors. *CrystEngComm* **14,** 5963 (2012).
6. Dubal, D. P., Gund, G. S., Lokhande, C. D. & Holze, R. Decoration of spongelike $Ni(OH)_2$ nanoparticles onto MWCNTs using an easily manipulated chemical protocol for supercapacitors. *ACS Appl. Mater. Interfaces* **5,** 2446–54 (2013).
7. Jiang, H., Li, C., Sun, T. & Ma, J. High-performance supercapacitor material based on $Ni(OH)_2$ nanowire-MnO_2 nanoflakes core–shell nanostructures. *Chem. Commun.* **48,** 2606 (2012).
8. Elshahawy, A. M. *et al.* Microwave – assisted hydrothermal synthesis of nanocrystal β-$Ni(OH)_2$ for supercapacitor applications. *CrystEngComm* **18,** 3256–3264 (2016).
9. Lee, D. U. *et al.* Self-Assembled NiO/$Ni(OH)_2$ Nanoflakes as Active Material for High-Power and High-Energy Hybrid Rechargeable Battery. *Nano Lett.* **16,** 1794–1802 (2016).
10. Wei, G. *et al.* Carbon quantum dot-induced self-assembly of ultrathin $Ni(OH)_2$ nanosheets: A facile method for fabricating three-dimensional porous hierarchical composite micro-nanostructures with excellent supercapacitor performance. *Nano Res.* **10,** 3005–3017 (2017).
